# Emotional and socio-cognitive processing in young children with symptoms of anxiety

**DOI:** 10.1007/s00787-022-02050-2

**Published:** 2022-07-21

**Authors:** Holly Howe-Davies, Christopher Hobson, Cerith Waters, Stephanie H. M. van Goozen

**Affiliations:** 1 School of Psychology, Cardiff University, Wales, UK; 2 Department of Clinical Child and Adolescent Studies, Leiden University, Leiden, Netherlands

**Keywords:** Anxiety, Theory of mind, Affective empathy, Cognitive empathy, Social relationships

## Abstract

Many children with anxiety disorders exhibit significant and persistent impairments in their social and interpersonal functioning. Two components essential for successful social interaction are empathy and theory of mind (ToM). Both constructs develop rapidly in childhood, but no study has simultaneously examined these skills in young children with emerging mental health problems, including those with symptoms of anxiety. This study investigated empathy and ToM in children with anxiety symptomatology and examined their relationship with anxiety severity. A cross-sectional study was carried out with 174 children aged 4–8 years with emerging mental health difficulties who were referred by school teachers for an assessment because of emotional, cognitive, or behavioural problems at school. Participants completed empathy and ToM tasks. Parents were interviewed and rated children’s emotional and behavioural problems. Correlational analyses indicated that elevated anxiety was associated with better cognitive ToM and worse affective empathy; there were no associations between anxiety and either cognitive empathy or affective ToM. Subsequent regression analyses demonstrated that whilst enhanced cognitive ToM was explained by age and verbal IQ, anxiety symptoms uniquely predicted impaired affective empathy. These results indicate that children with symptoms of anxiety have difficulty in sharing in other people’s emotions. As a result, they may find it difficult to behave in socially adequate ways in interactions with others that involve affective sharing. These findings encourage the use of early and targeted interventions that improve affective empathy development in children with anxiety symptoms.

**Supplementary Information:**

The online version contains supplementary material available at 10.1007/s00787-022-02050-2.

## Introduction

Anxiety disorders are some of the most common psychiatric disorders in childhood and adolescence. In children younger than 12 years, the prevalence varies between 2.6 and 5.2%, with separation anxiety being the most common disorder [1, 2]. Many children with anxiety disorders exhibit significant and persistent impairments in their social and interpersonal functioning [3]. It has been proposed that the prolonged negative affectivity that typifies anxiety may give rise to inflexible styles of processing emotional and socio-cognitive information [4], which can exacerbate these interpersonal problems. Having an understanding of one’s own and others’ mental states are socio-cognitive and emotional processes essential for successful social interaction [5, 6]. The present study sought to investigate the role of anxiety in children’s ability to understand their own and other children’s intentions, emotions, and behaviours.

### Empathy and theory of mind (ToM)

Empathy is generally defined as the ability to understand and share the feelings of another [7]. The ability to understand and appropriately respond to the feelings of others forms the basis for prosocial behaviour, social competence, and the maintenance of meaningful relationships [8, 9]. There is general agreement that empathy has both cognitive and affective components. Affective empathy refers to the ability to share the emotional experience of others (“I feel what you feel’) and respond with an appropriate emotion response, whilst cognitive empathy is the ability to model others’ emotional states (“I understand what you feel”) [10].

Impairments in empathy have been extensively documented in a range of childhood psychological disorders, including autism spectrum disorders [11], attention deficit/hyperactivity disorder [12] and disruptive behaviour disorders [13, 14]. Difficulties in understanding and responding to the emotional states of others appear to be directly related to the interpersonal and social difficulties that are prevalent in those with such disorders [15, 16]. Empathy impairments have also been associated with conduct problems and psychopathic traits in samples of pre-diagnostic children [17], suggesting that empathy impairments can be observed during children’s social development and prior to psychiatric disorder onset.

ToM involves the ability to make inferences about the psychological states (intentions, desires, beliefs, emotions) of others [18]. This enables an individual to understand and/or predict other people’s behaviour in social situations. Like empathy, ToM consists of two component processes, affective and cognitive ToM. The former refers to the ability to make inferences about other people's emotions, whilst the latter is the capacity to make assumptions about people's thoughts and beliefs [19]. The ability to develop accurate accounts of what people are thinking and feeling enables individuals to respond accordingly. Consequently, the development of ToM has important implications for children’s social communication, interaction, and behaviour [20].

The definition of affective ToM makes it synonymous to cognitive empathy. Indeed, some researchers argue that both affective and cognitive ToM form cognitive empathy, and thus ToM and cognitive empathy are conceptually interchangeable [21]. Neuroanatomical research [22] indicates involvement of cognitive (medial prefrontal cortex, superior temporal sulcus, temporal poles) and affective (ventromedial prefrontal cortex) ToM networks when a cognitive empathic response is generated. In contrast, affective empathic responses are driven mainly by regions that mediate emotional experiences, such as the amygdala and the insula.

A further distinction between affective empathy and the other three constructs relates to the self/other nature of inferences; whilst cognitive empathy, cognitive ToM and affective ToM are all concerned with inferring and understanding the mental states of others, affective empathy is associated with the ability to comprehend and communicate one’s own emotions. Neuroscientific and behavioural studies indeed suggest that mentalizing concepts can be organised around four dimensions or polarities, one of which is mentalizing about the self and about others [23].

### ToM, cognitive empathy, and anxiety

Theoretical models of the development of anxiety assume that impairments in social information processing play an important role [24]. Appraisal models of anxiety have hypothesised that anxious individuals exhibit attentional biases in relation to threat [25, 26] and biases in the interpretation of ambiguous material [27, 28]. As a result, children with anxiety may have difficulty in accurately interpreting social situations and, in turn, difficulty in understanding other people [29]. These children also tend to focus on their internal physiological cues, such as an increased heart rate [30, 31]. Because of this tendency to engage in excessive self-focussed attention and self-monitoring, children with anxiety may experience a reduced capacity to identify and understand the mental states of others. Indeed, there is evidence that social anxiety, panic disorder and separation anxiety in childhood are associated with impairments in ToM [32–35].

However, some children with anxiety may display advanced mental state recognition and understanding. For example, individuals with anxiety may be highly self-conscious, very sensitive to others’ opinions of them and have greater evaluative concerns [36]. These tendencies are associated with an excessive alertness to social signals and, in turn, advanced ToM and cognitive empathy [37, 38]. Supporting this perspective, one study conducted in 8- to 12-year-old children found that subclinical levels of social anxiety were related to greater accuracy in detecting mental states indirectly, through heightened self-consciousness [36].

Several issues should be considered when interpreting the mixed findings about the associations amongst anxiety and social cognition. First, a variety of tasks have been used to measure ToM. Not only do these tasks capture different aspects of ToM, such as the detection of faux pas [33], false belief understanding [32] and accuracy in detecting mental states from the eye region [36], but they also differ in their degree of complexity, level of arousal elicited and intensity of affect displayed. Each of these factors can moderate performance [39, 40].

Secondly, researchers often claim that young children have difficulties with the verbal-conversational aspects of many social cognition tasks [41]. Thus, children perform poorly not because they lack the requisite conceptual competence but rather because of the linguistic complexity of the task material [42]. As such, it is plausible that the relationship between anxiety and social cognition differs as a result of intellectual ability. Most of the aforementioned studies did not control for IQ or verbal ability in their analyses [32, 33, 36].

Finally, studies investigating the relationship between anxiety and social cognition in childhood have used samples ranging in age from toddlerhood to middle childhood. The relationship between anxiety and socio-cognitive processing may differ across time and development, so it would be beneficial to investigate the relationship at critical time points in the developmental trajectory. Early to middle childhood is the time when children begin to develop and enhance these important emotional and social cognitive skills, due to increases in social demands [43], and also when emotional problems emerge in those who are at risk and vulnerable. Therefore, it is important to investigate the early manifestations of anxiety and their relation to socio-cognitive abilities.

### Affective empathy and anxiety

Other theories of anxiety propose that anxiety is associated with dysregulated emotions [44], which may create an association with impaired affective empathy. If social cues are interpreted as threatening, individuals with anxiety will experience fear and elevated levels of physiological arousal [45]. Moreover, the greater evaluative concerns and alertness to social signals in anxious individuals are likely to increase their self-consciousness and emotional arousal [36, 46]. Consequently, children with anxiety may be less able to engage in adequate affective sharing and to respond appropriately in emotional situations.

Although there is no evidence of impaired affective empathy in anxious children, Morrison and colleagues [47] found that adults with social anxiety disorder had greater difficulty in vicariously sharing others’ positive emotions, compared to typically developing controls. Furthermore, indirect support for a link between anxiety and low affective empathy comes from research on alexithymia, where it has been shown that impairments in self-mentalising abilities are related to heightened anxiety [48, 49].

### The present study

We examined cognitive and affective empathy and cognitive and affective ToM performance in young children identified as at risk for mental health problems, including children demonstrating early anxiety symptoms. The first aim of the study was to characterise the relationship between childhood anxiety and different socio-cognitive and emotional processes. We hypothesised that anxiety symptomatology would be associated with impaired affective empathy. Given the lack of clarity about the nature of the association between anxiety and social cognition in children, we explored the relationship between anxiety and cognitive empathy and ToM. Secondly, we sought to examine whether anxiety severity could predict empathic performance, predicting that more severe anxiety symptoms would predict more severe affective empathy impairments. Because some studies have shown that empathy and ToM are influenced by gender and age [50, 51], we also explored the moderating role of gender and age in the any associations between anxiety and socio-emotional functioning.

## Methods

### Participants

Participants were referred to the Neurodevelopment Assessment Unit (NDAU; https://www.cardiff.ac.uk/neurodevelopment-assessment-unit) at Cardiff University by their teachers for an assessment because of emotional, cognitive, or behavioural difficulties at school. The NDAU is an assessment centre available to schools with concerns about a pupil’s functioning and the sample therefore demonstrated a heterogeneous range of difficulties, including children with low through to high levels of emotional and/or behavioural problems. None of the children had received a diagnosis at the time of testing, although many were on the diagnostic pathway. Written informed consent was obtained from the parent or caregiver for each child prior to the assessment and all experimental procedures were approved by the relevant university ethics committee (EC.16.10.11.4592GR).

The sample consisted of 174 children (58 female) aged 4–8 years (M = 6.27, SD = 1.08). We were unable to obtain complete data for the entire sample due to difficulties associated with the assessment of young children with emotional and behavioural problems. As a result, the numbers of children examined in individual analyses varied. However, a broadly similar percentage of complete data for each variable was collected in each of 5 age groups, as shown in Table 1, the only exception being the slightly lower proportions of complete empathy data for 4- to 5-year-olds.

**Table 1:** Number (and percentage) of participants in each of 5 age groups for whom complete measures of key constructs were available

Age (years)	Number of participants	Cognitive empathy	Affective empathy	Cognitive ToM	Affective ToM	Anxiety
4–5	23 (13.2%)	16 (70.0%)	16 (70.0%)	22 (95.5%)	21 (91.3%)	23 (100%)
5–6	47 (27.0%)	40 (85.1%)	40 (85.1%)	44 (93.6%)	44 (93.6%)	46 (97.9%)
6–7	56 (32.2%)	54 (96.4%)	54 (96.4%)	52 (92.9%)	52 (92.9%)	53 (94.6%)
7–8	40 (23.0%)	39 (97.5%)	39 (97.5%)	39 (97.5%)	39 (97.5%)	40 (100%)
8	8 (4.6%)	8 (100%)	8 (100.0%)	7 (87.5%)	7 (87.5%)	8 (100%)
Total	174 (100%)	157 (90.2%)	157 (90.2%)	164 (94.3%)	163 (93.7%)	170 (97.7%)

Percentages in column 2 are within-column; elsewhere the percentages are within-row

### Testing procedure

The child was tested individually by a trained graduate researcher and the tasks were administered in the same fixed order for each child. Simultaneously, in a separate room, parents completed a diagnostic interview and a battery of self-completion questionnaires regarding their child’s behaviour during the previous 6 months. The diagnostic interview (described below) comprised the Strengths and Difficulties Questionnaire and the Development and Wellbeing Assessment. A trained post-graduate researcher administered the online version of the diagnostic interview to parents and caregivers and provided verbal instructions for the self-completion questionnaires.

### Materials

#### Strengths and Difficulties Questionnaire (SDQ)

The Strengths and Difficulties Questionnaire (SDQ) is a 25-item screening questionnaire for emotional and behavioural problems in children and young people aged 3–16 years. Parents rated their child’s behaviour during the last 6 months on a 3-point Likert scale (0 = not true; 1 = somewhat true; 2 certainly true). The questionnaire consists of 5 subscales; four assess emotional and behavioural problems (emotional symptoms, conduct problems, hyperactivity/inattention and peer relationship problems) and one assesses positive behaviours (prosocial behaviour). In addition to subscale scores, the SDQ provides a Total Difficulties score, made up of the four emotional and behavioural problem subscale scores. The SDQ has been found to discriminate well between children with and without psychological problems [52, 53] and is a proven effective tool to screen for child psychiatric disorders, including anxiety problems, in community samples [54, 55].

The emotional subscale used in the main data analysis of the present study consists of 5 items tapping into anxiety (e.g. “Many fears, easily scared”, “nervous or clingy in new situations”), low mood (e.g. “Often unhappy, downhearted”) and psychosomatic symptoms (e.g. “Often complains of headaches”). Scores on the emotional subscale can range from 0 to 10, and scores on this subscale correlate significantly with anxiety symptom scores as assessed by other measures of childhood anxiety (e.g. the Revised Children’s Manifest Anxiety Scale) [56]. Overall, the sample showed a slightly elevated level of emotional symptoms, with 32% scoring ‘high’ or ‘very high’ on the SDQ emotional subscale.

Each SDQ subscale demonstrated acceptable internal consistency, although the reliability estimate for peer problems was slightly lower (Cronbach’s *α*s: emotional problems = 0.75; conduct problems = 0.78; hyperactivity = 0.77; peer problems = 0.60; prosocial = 0.79).

#### The development and well-being assessment

The Development and Wellbeing Assessment (DAWBA) is an in-depth diagnostic interview used to assess children’s psychopathology according to DSM-IV-TR [57] and ICD-10 [58] taxonomy. The current study used the online parent version of the DAWBA suitable for primary school-aged children [55]. The online version of the DAWBA first takes respondents through the aforementioned SDQ, which acts as a screening questionnaire, before moving on to the more detailed DAWBA interview that covers a wide range of specific diagnoses. Upon completion, the DAWBA scoring system proposes likely diagnoses based on the parent ratings, ranging from a probability of less than 0.1% of having the relevant diagnosis to a probability of over 70% of having the relevant diagnosis. The DAWBA reliably and validly discriminates between community and clinic samples in rates of diagnosed disorder [55] and has been widely used in research on child and adolescent mental health [59].

Scores for social anxiety, separation anxiety and generalised anxiety data were used in the present study. In addition to producing DSM-IV-TR and ICD-10 orientated diagnoses, dimensional scores for each disorder can be calculated. The dimensional scores for separation anxiety, social anxiety and generalised anxiety are based on the total number of worry areas (e.g. worries about past behaviour, worries about sleeping alone), the total number of symptoms (e.g. worrying leads to concentration difficulties) and the severity of symptoms (a little/a lot). Parents’ ratings on the SDQ and DAWBA were used because, for younger children, parents are the best informants in rating internalising disorders [60].

#### Intellectual ability

To assess cognitive ability, the Lucid Ability assessment was administered [61]. The Lucid estimates full-scale IQ (FSIQ), verbal IQ and performance IQ. For children aged 4–6 years verbal IQ is assessed by a picture vocabulary task, and performance IQ by a mental rotation task. For older children, aged 7–16 years, verbal IQ is assessed via a conceptual similarities task, and performance IQ through a matrix problem-solving task. An overall measure of FSIQ is calculated based on the sum of verbal and performance IQ scores. The validity of Lucid Ability is comparable to the Wechsler Intelligence Scale for Children (WISC-III), the British Ability Scales (Second Edition) and the British Picture Vocabulary Scale (Second Edition) [61, 62].

#### Measurement of empathy

Participants viewed three empathy-inducing film clips. The three clips were used in a study by Noten and colleagues [63], who demonstrated that three-year-old children were able recogni*s*e and understand the emotions presented in these videos. One clip represented happiness (a boy opening a Christmas present), another sadness (a boy flushing his dead goldfish down the toilet), and another one fear (a girl afraid of being in a car wash). Following each clip, children were asked questions about the emotions of the main character and about their own emotions whilst viewing the clip. They were asked how strongly they and the main character felt each of six emotions (affective empathy) and to explain the reason for the emotion (cognitive empathy). A coding system adapted from Strayer's [64] empathy continuum was used to score children’s affective and cognitive responses (for details on cognitive empathy scoring, see Braaten and Rosén [65]; for details on affective empathy scoring, see Strayer [64]). Affective empathy scores ranged from 0 to 6 for each clip and cognitive empathy scores from 0 to 8 for each clip, with higher scores reflecting greater empathy. No participants achieved the highest possible cognitive and affective empathy scores, suggesting that ceiling effects were not present [66].

A randomly selected subset of transcripts (15%) was independently coded by two trained coders who were blind to any additional details of the child; the interrater agreement was 92.3% and inter-scorer reliabilities (Cohen’s kappa) between the two coders ranged from 0.82 (cognitive) and 0.98 (affective).

#### ToM comprehension

ToM understanding was assessed with a four-item false belief (FB) battery, including three cognitive tasks and one affective task. The first cognitive item was an adaptation of Wellman and Lui’s [67] *unexpected contents task*, also known as the *contents FB task*. This involved showing the child a tube of ‘Smarties’ with pencils inside and asking the child what another person would think is in the tube: ‘Smarties’ or pencils. *The changed location task*, similar to Baron-Cohen and colleagues’ [68] explicit FB measure, involved a story about a boy, Max, who puts his football in a covered basket and then goes outside to play. Whilst Max is outside, Sally moves the football to a box. The child was then asked where Max would look for his football. The third cognitive task was an adapted version of Coull, Leekam, and Bennett [69] and Perner and Wimmer’s [70] *second-order false FB task*. This task involved a story about a boy named Nick, who hid his teddy in his bed. When Nick leaves the room, Alex takes the teddy and hides it in the cupboard. Nick comes back and sees Alex hiding the teddy, but Alex doesn’t see Nick. The child participant was asked where Alex would think Nick would look for his teddy. The affective task was an emotion FB test, known as the *Belief-Emotion Task*, which involved showing the child a ‘Coco-pops’ box with rocks inside. The child was then introduced to Teddy who loves Coco-pops and asked how Teddy feels when he sees the box. This task is based on one used by Wellman and Lui [67]. A fixed presentation order was used for the four tasks: (1) belief-emotion, (2) explicit FB, (3) contents FB, and (4) second-order FB.

All tasks consisted of test questions and memory questions. If participants answered all questions correctly for the task, they were awarded one point (score range: 0–4). The belief-emotion, contents FB and second-order FB tasks also included a justification question (e.g. why does Tiger think there are Smarties in the tube?). These questions resulted in the award of 2, 1 or 0 points, depending on the correctness of the mental state terms spontaneously used by the child (score range: 0–6). No justification question was asked in relation to the explicit false belief task, to prevent carry-over effects with the later administered second-order task. (See Table S1 in Supplementary Materials for more information of the coding of mental state terms).

A total ToM score, ranging from 0 to 10, consisted of the sum of scores on the four ToM items; a total affective ToM score, ranging from 0 to 3, consisted of the score on the emotion FB task; and a total cognitive score, ranging from 0 to 7, consisted of the sum of scores on the unexpected contents FB, the explicit FB and the second-order FB tasks. Seven per cent of participants achieved the highest possible affective ToM score, whilst no participants achieved the highest cognitive score, again suggesting that ceiling effects were not present [66].

A randomly selected subset of transcripts (15%) was independently coded by two trained coders who were blind to any additional details about the child; the interrater agreement was 90.7% and the inter-scorer reliabilities (Cohen’s kappa) between the two coders ranged from 0.68 to 1.00 (mean κ = 0.86).

### Statistical analysis

Because the SDQ emotional subscale is a very brief screening measure for internalising symptoms, its scores were combined with the DAWBA anxiety dimensional scores (social anxiety, separation anxiety and generalised anxiety DAWBA dimensional scores combined) to create a composite anxiety score. SDQ emotional subscale scores correlated positively with DAWBA anxiety dimensional scores (*r* = 0.74, *n* = 170, *p* ≤ 0.001, *d* = 0.55, 95% CI [0.67, 0.80]). To assess the relationships between anxiety and ToM, and anxiety and empathy, Pearson’s correlations were calculated between anxiety composite scores on one hand, and cognitive ToM, affective ToM, cognitive empathy and affective empathy task scores, on the other. We then conducted two sets of moderated hierarchical regression analyses, first to assess the unique effects of anxiety on ToM and empathy, and then to examine whether the expected relationships between ToM and anxiety and empathy and anxiety were moderated by age and gender.

In the first regression model, affective empathy was entered as the dependent variable (DV). In the second model, cognitive ToM was entered as the DV. Predictor variables were entered into each of the regression equations in 3 steps. Age, gender, FSIQ, conduct problems, peer problems and prosocial behaviour covariates were entered at step 1, and anxiety was entered at step 2. Variance Inflation Factors (VIFs) were examined to assess whether variance in the resulting beta coefficients were inflated due to multicollinearity. A VIF larger than 5 is an indication of multicollinearity [71]. VIFs ranged from 1.02 for FSIQ to 1.43 for SDQ prosocial scores, indicating that multicollinearity was not an issue. The interactions between anxiety and age and anxiety and gender were then entered at Step 3 in both models. Interaction terms were created by calculating the product of the centred main effect (anxiety) and gender (scored as 0 = male; 1 = female) and by calculating the product of the centred main effect and a centred age variable.

All statistical analyses were performed using the Statistical Package for the Social Sciences (IBM SPSS 24) software and significance level was set at α < 0.05.

## Results

### Preliminary analyses

Table 2 displays descriptive data for intellectual ability, SDQ scores, DAWBA anxiety dimensional scores and empathy and ToM task performance. Positive relationships were identified between anxiety and parent-rated peer problems [*r* = 0.22, *n* = 170, *p* = 0.005, *d* = 0.05, 95% CI 0.07, 0.36]] and conduct problems [*r* = 0.17, *n* = 170, *p* = 0.027, *d* = 0.03, 95% CI [0.02, 0.31]]. Anxiety scores were negatively related to prosocial behaviour [*r* = −0.19, *n* = 170, *p* = 0.016, *d* = −0.03, 95% CI [−0.33, −0.04]]. There was no difference in anxiety symptomatology between girls and boys, nor were there significant associations between anxiety and age or intellectual ability. Positive relationships were also identified between cognitive empathy and affective empathy [*r* = 0.69, *n* = 157, *p* < 0.001, *d* = 0.48, 95% CI [0.60, 0.76]], between cognitive empathy and cognitive ToM [*r* = 0.20, *n* = 149, *p* < 0.05, *d* = 0.04, 95% CI [0.04, 0.35]] and between cognitive ToM and affective ToM [*r* = 0.38, *n* = 162, *p* < 0.001, *d* = 0.14, 95% CI [0.23, 0.50]]. There were no significant associations between cognitive empathy and affective ToM, or between affective empathy and either affective or cognitive ToM (see Table S2 in Supplementary Materials).

**Table 2:** Descriptive data for intellectual ability, SDQ scores, DAWBA anxiety dimensional scores and empathy and ToM task performance

	Mean (SD)	Range
FSIQ	98.83 (11.86)	75–137
*Empathy*		
Cognitive empathy	9.84 (4.79)	3–21
Affective empathy	7.53 (4.00)	0–16
*ToM*		
Cognitive ToM	2.32 (1.86)	0–6
Affective ToM	1.37 (1.01)	0–3
*SDQ*		
SDQ Emotional	3.65 (2.68)	0–10
SDQ Conduct	4.15 (2.67)	0–10
SDQ Peer	2.99 (2.25)	0–9
SDQ Hyperactivity	7.75 (2.52)	0–10
SDQ Prosocial	6.71 (2.46)	0–10
DAWBA anxiety	11.63 (14.68)	0–68

DAWBA Anxiety measure consists of separation anxiety, social anxiety and generalised anxiety dimensional scores; FSIQ, full-scale intelligence quotient; SDQ, Strengths and Difficulties Questionnaire. FSIQ N = 169. Empathy N = 157. ToM = 164. SDQ = 170. DAWBA = 170

Prior to testing study hypotheses, preliminary analyses investigated differences between male and females on all socio-cognitive and emotional variables, in addition to the variables’ relations with age. Results showed that boys had lower cognitive empathy [*t*(155) =  −2.693, *p* = 0.008, *d* = −0.45, 95% CI [−3.65, −0.56]], affective empathy [*t*(155) =  −2.253, *p* = 0.026, *d* = −0.38, 95% CI [−2.78, −0.18], and cognitive ToM [*t*(155) =  −2.425, *p* = 0.016, *d* = −0.40, 95% CI, [−1.32, −0.14]] scores than girls. Similarly, age was positively related to cognitive empathy [*r* = 0.30, *n* = 157, *p* < 0.001, *d* = 0.01, 95% CI [0.15, 0.44]], affective ToM [*r* = 0.23, *n* = 163, *p* = 0.003, *d* = 0.05, 95% CI [0.08, 0.37]] and cognitive ToM scores [*r* = 0.47, *n* = 164, *p* < 0.001, *d* = 0.22, 95% CI [0.34, 0.58]].

#### Main analyses

Anxiety was significantly positively related to cognitive ToM performance [*r* = 0.16, *n* = 164, *p* = 0.048, *d* = 0.02, 95% CI [0.00, 0.30]] and significantly negatively related to affective empathy [*r* = -0.19, *n* = 153, *p* = 0.020, *d* = 0.04, 95% CI [−0.34, −0.03]]. There were no significant associations between anxiety scores and either cognitive empathy or affective ToM.

Hierarchical multiple regression analyses were conducted to determine the relative contribution of anxiety symptoms to the explanation of affective empathy and cognitive ToM performance. The moderating roles of gender and age were also investigated by examining their interactions with anxiety.^1^

#### Affective empathy

At step 1, the overall model was not significant, *R*^2^ = 0.030, *F*(6, 142) = 0.74, *p* = 0.619. The addition of anxiety in step 2 of the model led to a significant change in *R*^2^ of 0.063, *F*(1, 141) = 9.80, *p* = 0.002, and showed that anxiety symptomatology uniquely predicted affective empathy. The interactions entered in Step 3 did not account for a significant increase in the proportion of explained variance in affective empathy, ∆*R*^2^ = 0.004, *F*(2, 139) = 0.30, *p* = 0.743. Full details for both regression models are presented in Table 3.

**Table 3:** Moderated hierarchical regression analysis model predicting affective empathy and cognitive ToM performance from anxiety symptomatology

Variable	Affective empathy						Cognitive ToM					
	Step 1		Step 2		Step 3		Step 1		Step 2		Step 3	
	*B*	*β*	*B*	*β*	*B*	*β*	*B*	*β*	*B*	*β*	*B*	*β*
Constant	6.75		5.94		6.54		−8.43		−8.36		−8.20	
Age	0.00	0.00	0.01	0.04	0.01	0.04	0.09	0.58***	0.09	0.57***	0.09	0.57***
Gender	1.23	0.15	1.52	0.19*	1.51	0.18*	0.59	0.15*	0.56	0.15*	0.56	0.14*
FSIQ	0.00	−0.01	0.00	0.01	0.00	0.00	0.04	0.27***	0.04	0.27***	0.04	0.26***
Conduct problems	0.12	0.08	0.16	0.10	0.16	0.10	0.01	0.01	0.00	0.00	0.00	0.00
Peer problems	0.10	0.05	0.15	0.08	0.15	0.08	0.01	0.01	0.03	0.00	0.00	0.00
Prosocial problems	0.03	0.02	−0.03	−0.02	−0.02	−0.01	−0.04	−0.05	−0.03	−0.04	−0.03	−0.04
Anxiety			−0.06	−0.26**	−0.08	−0.33**			0.01	0.06	0.00	0.03
Anxiety × age					0.13	0.03					−0.04	−0.02
Anxiety × gender					0.40	0.07					0.16	0.06
*R*²	0.03		0.09		0.10		0.31		0.31		0.31	
*F*	0.74		9.80**		0.30		11.22***		0.69		0.18	

^*^ *p* < .05, ** *p* < .01 *** *p* < .001. B = unstandardized regression coefficient. β = standardised coefficient

Anxiety measure consists of combined DAWBA and SDQ Emotional dimensional scores. Affective empathy model *N* = 149; cognitive ToM model *N* = 159

#### Cognitive ToM

At step 1, the overall model was significant, *R*^2^ = 0.307, *F*(6, 152) = 11.22, *p* < 0.001; age, gender and FSIQ uniquely predicted cognitive ToM performance. The addition of anxiety to the model did not lead to a significant change in explained variance, ∆*R*^2^ = 0.003, *F*(1, 151) = 0.69, *p* = 0.409. The addition of the interaction terms at step 3 also did not add significantly to the explained variance, ∆*R*^2^ = 0.002, *F*(2, 149) = 0.18, *p* = 0.835 (see Table 3).

## Discussion

The current study focussed on the role of anxiety in empathy and theory of mind (ToM) performance in young children identified as being at risk for mental health problems; this sample included children demonstrating early anxiety symptoms. Correlational analysis identified the presence of a unique social profile in relation to childhood anxiety: anxiety was positively correlated with cognitive ToM, but negatively correlated with affective empathy. However, the results of regression analyses demonstrated that, after controlling for age, gender, and IQ, severity of anxiety only predicted lower affective empathy.

In line with our theoretically informed predictions, the results show that affective empathy is negatively related to anxiety. Morrison et al. [47] similarly reported impairments in affective empathy for positive emotions in a sample of adults with social anxiety disorders. Our finding of reduced affective empathy is consistent with theoretical frameworks proposing the dysregulation of emotions in anxious children [44]. Children with anxiety experience fear, heightened self-consciousness, and high levels of physiological arousal [36, 46], which may limit their capacity to share in other people’s emotions. An inability to engage in affective sharing may, in turn, result in children with anxiety behaving in socially inadequate ways in response to emotive situations. There is empirical evidence that arousal beyond a certain level interferes with an individual’s ability to respond adaptively in emotionally evocative situations [72]. Elaborating on these results, if children with anxiety respond to others’ emotions inappropriately on a regular basis this could have detrimental effects on their interpersonal relationships. Indeed, children with anxiety disorders have been reported to have lower levels of peer acceptance, relative to typically developing controls [73]. This was also observed in the current study, where higher levels of anxiety problems were associated with higher levels of parent-rated peer problems.

We predicted that affective empathy performance would be influenced primarily by anxiety symptomology. Regression analysis confirmed that severity of anxiety symptoms uniquely predicted affective empathy impairments, over and above the influence of age, gender, IQ, conduct problems, peer problems and prosocial behaviour. These findings support the idea that impairments in affective empathic responding are important in childhood anxiety and therefore point to the importance of early and targeted interventions.

Our study also aimed to understand the relationships between anxiety and both ToM and cognitive empathy, hypothesising that there would be an association between anxiety, on the one hand, and affective ToM, cognitive ToM and cognitive empathy, on the other. Although we found a positive relationship between cognitive ToM and anxiety, regression analysis showed that cognitive mentalising ability was predicted by age and IQ, rather than severity of anxiety symptoms. Contrary to expectations, we found no evidence of a relationship between anxiety symptom severity and either cognitive empathy or affective ToM. These results are inconsistent with previous studies that have observed either enhanced [36] or impaired [32–35] abilities in theorising about others’ mental states in children with anxiety. These discrepancies in findings may arise from variation in a number of task-related factors. For example, socio-cognitive research has found that the type of social cognition task used, the level of arousal elicited by tasks, the intensity of affect displayed in facial emotion, and task complexity can all moderate performance on these tasks [39, 40]. Indeed, Banerjee and Henderson [33] found that whilst children with and without social anxiety did not differ in their performance on second-order false belief tasks, children with social anxiety performed significantly worse on the faux pas task than did controls. The faux pas task requires an advanced understanding of the links between emotions, intentions and beliefs in social situations, leading the authors to suggest that children with social anxiety only demonstrate impairments in advanced ToM skills. However, it is also possible that the relationship between mentalizing and anxiety observed by Banerjee and Henderson [33] was a reflection of task complexity. Therefore, the association could be explained in terms of IQ or verbal abilities, rather than psychopathology, as evidenced in the current study.

The lack of an association between anxiety and cognitive empathy and ToM is also inconsistent with social information processing theories. Theoretical models propose that the relation between cognition and emotion is one of interdependence. Just as cognition can influence emotion [24], emotion can also shape cognition [74]. For example, attentional bias during encoding can evoke fear and related physiological responses; but equally, fear can create attentional bias [75]. Such bias, in turn, is likely to disrupt children's processing of socio-cognitive information and as a result exacerbate the interpersonal dysfunction exhibited by children with anxiety disorders. The fact that children's anxiety was negatively related to affective empathy but unrelated to cognitive theory of mind (after controlling for age and IQ) might therefore be regarded as surprising. If anxiety has a disruptive effect on affective empathy, why was it unrelated to a measure of socio-cognitive processing? The answer may lie in the very different nature of the stimulus material used in the tasks measuring affective empathy and cognitive theory of mind. Affective empathy was measured using real life videos depicting another child experiencing emotions. It may be the case that anxious children do worse when presented with such videos because the combination of their own dispositional arousal and arousal evoked by the stimulus video makes it more difficult for them to identify what the target child is experiencing. By comparison, the stimulus material used in cognitive theory of mind task is predominantly verbal and considerably less arousing in nature. Here, it may be that anxiety neither disrupts nor benefits task performance.

### Strengths, limitations, and future research

The current study has several limitations. First, given the nature of our design, we are unable to draw firm conclusions about the direction of the observed relationship. We proposed that the more frequent and more intense negative affectivity that typifies those with anxiety symptoms may give rise to an inflexible style of processing socio-cognitive and emotional information [4], which in turn, may exacerbate the interpersonal problems exhibited by children with anxiety disorders. However, there is also evidence that impairments in social cognitive processing (i.e. cognitive biases, attentional biases) and more intense and dysregulated emotional experiences in early childhood can lead to higher levels of anxiety [46, 76]. Confirmation of the directionality of the relationship remains to be investigated. Second, this study explored only one component of ToM, namely false belief. Whilst the development of false belief understanding is central between the ages of 4 and 7, and assessing this component of ToM provided us with information on impairments that are highly representative of children in this age group, ToM also involves understanding of others’ intentions and desires [67]. A third limitation concerns the use of a single vignette to assess happiness and sadness to evaluate affective ToM reasoning. It would be interesting to establish whether children with anxiety symptomatology exhibit intact affective ToM when presented with tasks assessing their understanding of more complex emotional displays. A final limitation is that complete data were not available for the entire sample, due to difficulties associated with assessing young children with emotional and behavioural problems (for example, some children with attentional difficulties were unable to complete all tasks).

Despite these limitations, the current study advances our understanding of the emotional and socio-cognitive processes that underlie elevated anxiety in young children. To our knowledge, this is the first study to examine affective and cognitive empathy, and affective and cognitive ToM in a sample of primary school-aged children who were identified by teachers as displaying emerging emotional and behavioural problems. This enabled the investigation of ToM and empathic abilities at a critical stage in socio-cognitive and emotional development and when emotional and behavioural problems have not yet reached ‘crisis point’. In turn, this facilitated a better understanding of the potential role of anxiety in the disruption of emotional processing, highlighting the importance of early intervention.

A further strength of the current sample is that all participants were referred to our centre for a range of often overlapping emotional, cognitive and behavioural problems. Evidence has consistently demonstrated a high rate of comorbidity for individuals presenting with anxiety disorders [77]. Children and adolescents with anxiety disorders have comorbid attention deficit hyperactivity disorder (15–50%), depression (25–50%) and/or disruptive disorders (20–63%) [77–79]. Consequently, our sample is highly representative of populations at risk of anxiety.

## Conclusion and clinical implications

Childhood anxiety is negatively associated with general well-being, social functioning and academic performance [80–82]. Prospective longitudinal studies have demonstrated that anxiety that develops in early childhood may be a risk factor for future mental illness in adolescence and adulthood [83, 84]. The current results indicate that impairments in affective empathy are already present in young children with anxiety symptomatology and that severity of anxiety predicts the intensity of these affective empathy impairments. It is possible that anxious children’s limited capacity to share in other peoples’ emotions is a result of the dysregulated social emotions and physiological arousal they experience. These findings highlight the need to encourage the development and use of early interventions for children that target these emotional processes. Existing evidence-based cognitive–behavioural therapy (CBT) interventions for children with anxiety could be adapted to include an emotion regulation component. For example, emotion-focussed CBT treatment protocols have been shown to be effective in improving emotion regulation skills and reducing anxiety symptoms in children diagnosed with anxiety disorders [85, 86].

### Supplementary Information

Below is the link to the electronic supplementary material.

Supplementary file1 (DOCX 14 KB)

## Footnotes

1 We ran similar analyses using cognitive empathy and affective ToM as outcome variables, to check whether their non-significant zero-order associations with anxiety concealed underlying moderation by gender or age. No significant effects were found, so these analyses are not reported here.
