# Supplementary material for: Emotional and socio-cognitive processing in young children with symptoms of anxiety
Source: Eur Child Adolesc Psychiatry. 2022 Jul 21;32(10):2077–88. doi: 10.1007/s00787-022-02050-2 (PMC10533571; doi:10.1007/s00787-022-02050-2)
Supplement: Supplementary file 1 — Supplementary file1 (DOCX 14 KB) [file 787_2022_2050_MOESM1_ESM.docx]

**Table S1** Scheme for coding ToM tasks

| **Unexpected Contents FB** |
| --- |
| 0 = answered test questions incorrectly |
| 1 = answered test questions correctly but did not provide an appropriate justification (*e.g., Because he loves them)*  2 = answered test questions correctly and provided an appropriate non-belief justification e.g., |
| *The misleading appearance (e.g., It’s a Smarties tube)* |
| *Non-belief mental state (e.g., He didn’t see)* |
| *Deception (e.g. He was tricked)*  3 = answered test questions correctly and provided an appropriate belief-based justification *(e.g., Because he doesn’t know what’s really in there)* |
| **Explicit FB** |
| 0 = answered test questions incorrectly |
| 1 = answered test questions correctly |
| **Belief-Emotion** |
| 0 = answered test questions incorrectly |
| 1 = answered test questions correctly but did not provide an appropriate justification *(e.g., Because he feels happy)* |
| 2 = answered test questions correctly and provided an appropriate non-belief justification e.g., |
| *Apparent contents (e.g., Because he has coco-pops)* |
| *Non-belief mental state (e.g., Because he hasn’t seen what’s inside)* |
| *Deception (e.g., Someone tricked him)* |
| *Preference (e.g. He likes coco-pops)* |
| 3 = answered test question correctly and provided an appropriate belief-based justification (*e.g., Because he thinks there’re coco-pops inside)* |
| **Second-Order FB** |
| 0 = answered test questions incorrectly |
| 1 = answered test questions correctly but did not provide an appropriate justification  2 = answered test questions correctly and provided an appropriate non-belief justification e.g., |
| *Original location of object is mentioned (e.g., Because that’s where he hid it)* |
| 3 = answered test question correctly and provided an appropriate belief-based justification, e.g., |
| *Nesting of crucial information within another’s belief (e.g., Because she hid in the cupboard, and then, and she thinks Nick didn’t see her)* |
| *Embedding of mental state (e.g., Because she thinks Nick thinks it is in the bed)* |

**Table S2** Pearson’s correlations (r) between cognitive empathy, affective empathy, cognitive ToM and affective ToM

|  | Cognitive empathy | Affective empathy | Affective ToM | Cognitive ToM |
| --- | --- | --- | --- | --- |
| Cognitive empathy | - | - | - | - |
| Affective empathy | .693** | - | - | - |
| Affective ToM | .093 | .031 | - | - |
| Cognitive ToM | .198* | -.125 | .375** | - |

* *p* < .05, ** *p* < .01. Empathy N = 157. ToM = 164.
